# Amylopectin Partially Substituted by Cellulose in the Hindgut Was Beneficial to Short-Chain Fatty Acid Production and Probiotic Colonization

**DOI:** 10.1128/spectrum.03815-22

**Published:** 2023-04-10

**Authors:** Yu Bai, Yaowen Zhang, Zhenyu Wang, Yu Pi, Jinbiao Zhao, Shujun Wang, Dandan Han, Junjun Wang

**Affiliations:** a State Key Laboratory of Animal Nutrition, College of Animal Science and Technology, China Agricultural University, Beijing, China; b State Key Laboratory of Food Nutrition and Safety, College of Food Science and Engineering, Tianjin University of Science & Technology, Tianjin, China; c Key Laboratory of Feed Biotechnology of the Ministry of Agriculture and Rural Affairs, Feed Research Institute, Chinese Academy of Agricultural Sciences, Beijing, China; Jilin University

**Keywords:** amylopectin fermentation, mixture of amylopectin and cellulose, ileal infusion, intestinal microbiota, short-chain fatty acids

## Abstract

Undigested amylopectin fermentation in the hindguts of humans and pigs with low digestive capacity has been proven to be a low-efficiency method of energy supply. In this study, we researched the effects and mechanisms of amylopectin fermentation on hindgut microbiota and metabolite production using an *in vitro* fermentation trial and ileal infusion pigs model. In addition, we also researched the effects of interaction between amylopectin and cellulose during hindgut fermentation in this study. Our results showed that amylopectin had higher short-chain fatty acid (SCFA) production and dry matter digestibility (DMD) than cellulose but was not significantly different from a mixture of amylopectin and cellulose (Amycel vitro) during *in vitro* fermentation. The Amycel vitro group even had the highest reducing sugar content and amylase activity among all groups. The ileal infusion trial produced similar results to vitro fermentation trial: the mixture of amylopectin and cellulose infusion (Amycel vivo) significantly increased the levels of reducing sugar, acetate, and butyrate in the hindgut compared with the amylopectin infusion (Amy vivo). The mixture of amylopectin and cellulose infusion also resulted in increased Shannon index and probiotic colonization in the hindgut. The relative abundance of *Romboutsia* in the Amycel vivo group, which was considered a noxious bacteria in the Amycel vivo group, was also significantly lower than that in the Amy vivo group. In summary, the high level of amylopectin fermentation in the hindgut was harmful to intestinal microbiota, but amylopectin partially substituted with cellulose was beneficial to SCFA production and probiotic colonization.

**IMPORTANCE** A high-starch (mainly amylopectin) diet is usually accompanied by the fermentation of undigested amylopectin in the hindgut of humans and pigs with low digestive capacity and might be detrimental to the intestinal microbiota. In this research, we investigated the fermentation characteristics of amylopectin through an *in vitro* fermentation method and used an ileal infusion pig model to verify the fermentation trial results and explore the microbiota regulatory effect. The interaction effects between amylopectin and cellulose during hindgut fermentation were also researched in this study. Our research revealed that the large amount of amylopectin fermentation in the hindgut was detrimental to the intestinal microbiota. Amylopectin partially substituted by cellulose was not only beneficial to antioxidant ability and fermentation efficiency, but also promoted SCFA production and probiotic colonization in the hindgut. These findings provide new strategies to prevent intestinal microbiota dysbiosis caused by amylopectin fermentation.

## INTRODUCTION

The hindgut is an important component of the gastrointestinal tract and the main site for microbe colonization. Microbes in the hindgut play important roles in animal growth and development and have also demonstrated a high potential to prevent diseases, such as intestinal inflammation, cardiometabolic disorders, and cancer (1, 2). Hindgut microbe degrade undigested nutrients into metabolites, including hydrogen, carbon dioxide, methane, and short-chain fatty acid (SCFA) like acetate, propionate, and butyrate (3). Acetate and propionate have been reported to have a strong ability to regulate energy metabolism and are involved in lipogenesis and gluconeogenesis (4). Butyrate is helpful for ameliorating intestinal inflammation and serves as the energy source for colonic mucosa cells (5). SCFA in the hindgut even contribute to 10% of the energy requirement in humans and 13% of that in pigs (6).

Exogenous nutrients are the major energy sources for intestinal microbe growth and activity, deeply influencing microbiota and metabolite production. Starch in the diet is the main energy sources for human and animal growth, including amylopectin (70% to 80%) and amylose (20% to 30%) (7). Recently, high-starch (mainly amylopectin) diet has been applied to humans and animals because of its good palatability and high production efficiency. However, a high-starch content diet is always accompanied by the fermentation of undigested amylopectin in the hindgut (8). In addition, soluble, high-viscosity fiber in diets could also cause low ileal starch digestibility and a high level of amylopectin fermentation in the hindgut: the amylopectin becomes wrapped or bound to fiber, preventing contact between amylopectin and amylase, which could be the main cause of these effects (9, 10).

Amylopectin fermentation is an inefficient means of energy supply, but its effects on hindgut microbiota and metabolite production are not well known. Cellulose is the most abundant fiber in the world and has been proven to have positive effects on microbiota regulation (11). We assumed that a large amount of amylopectin in the hindgut is harmful to intestinal microbiota and metabolite production, and that partial substitution of amylopectin by cellulose might be beneficial to hindgut fermentation.

The purpose of this study was to investigate the effects and mechanisms of amylopectin fermentation on intestinal microbiota and metabolite production in the hindgut by *in vitro* fermentation method and the ileal infusion pigs model. The interaction effects between amylopectin and cellulose during hindgut fermentation were also researched in this study.

## RESULTS

### DMD, reducing sugar content and SCFA production during *in vitro* fermentation.

**FIG 1:**
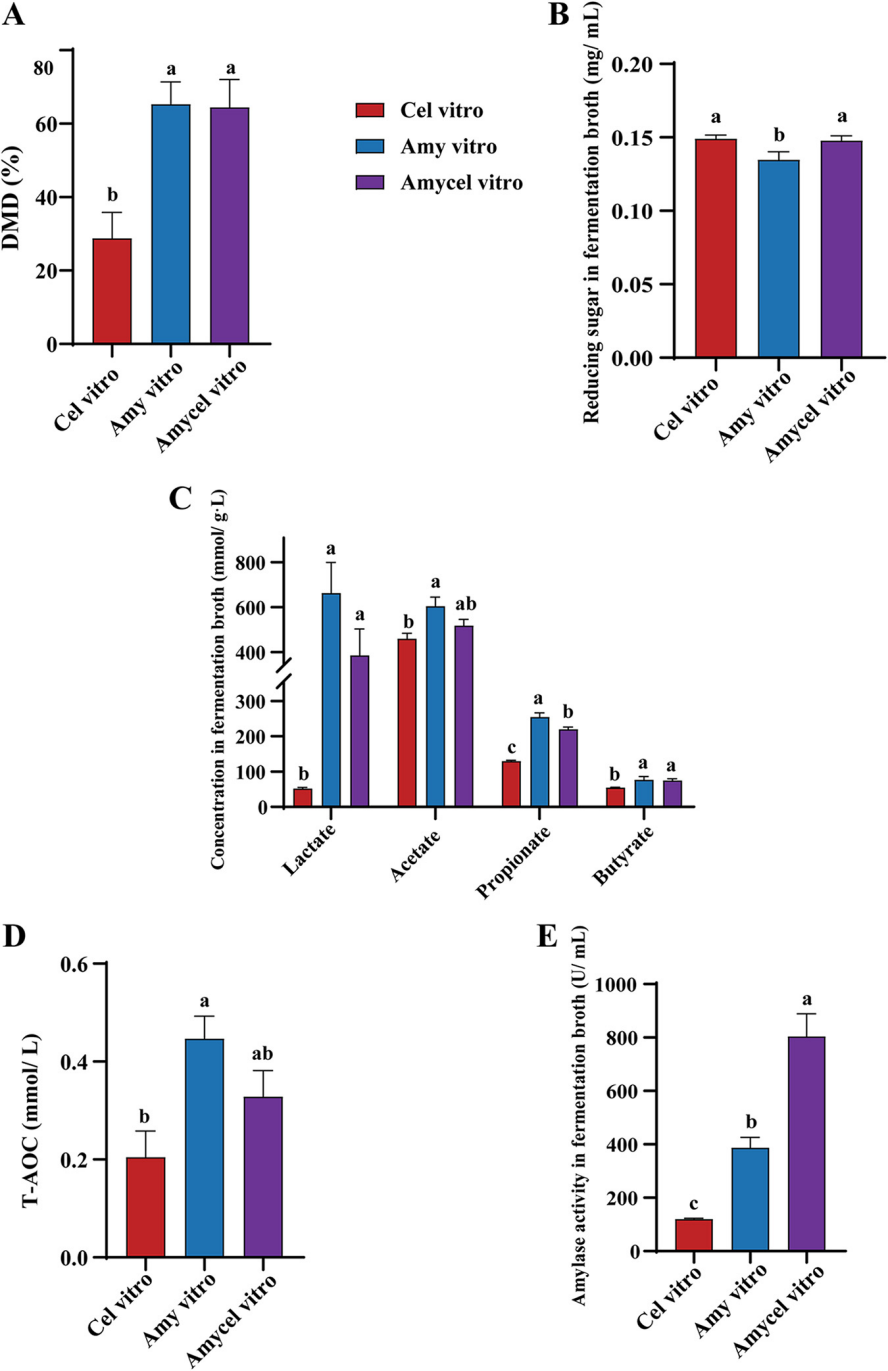
(A) The dry matter digestibility (DMD) of three groups during *in vitro* fermentation. (B) Reducing sugar content in the fermentation broth. (C) Concentration of lactate, acetate, propionate, and butyrate in the fermentation broth. (D) Total antioxidant capacity (T-AOC) in the fermentation broth. (E) Amylase activity in the fermentation broth. Results are shown as means ± standard error of the mean (SEM), *n* = 6. Different lowercase letters indicate significant differences, *P < *0.05 (one-way analysis of variance [ANOVA] with Duncan). Cel vitro, cellulose vitro fermentation; Amy vitro, amylopectin vitro fermentation; Amycel vitro, the mixture of amylopectin and cellulose vitro fermentation.

The dry matter digestibility (DMD) of the Amy vitro (amylopectin vitro fermentation) and Amycel vitro groups (mixture of amylopectin and cellulose vitro fermentation) were higher (*P < *0.05) than that of the Cel vitro group (cellulose vitro fermentation) (Fig. 1A), and there was no significant difference in DMD between them (Fig. 1A). The reducing sugar content of the Amy vitro group was lower (*P < *0.05) than that of the Amycel vitro and Cel vitro groups (Fig. 1B). Although lactate, acetate, and butyrate were lower in the Cel vitro group (*P < *0.05) than in the Amy vitro group, there were no significant difference between the Amy vitro and Amycel vitro groups (Fig. 1C).

### Total antioxidant capacity and amylase activity during *in vitro* fermentation.

The Amy vitro group had a higher (*P < *0.05) total antioxidant capacity (T-AOC) than the Cel vitro group, but the T-AOC level of the Amycel vitro group was not significantly difference from that of the Amy vitro group (Fig. 1D). Amylase activity was also higher in the Amy vitro group than in the Cel vitro group, but the Amycel vitro group had the highest amylase activity among all groups (*P < *0.05) (Fig. 1E).

### SCFA production, reducing sugar content, and amylase activity in the hindgut of growing pigs.

**FIG 2:**
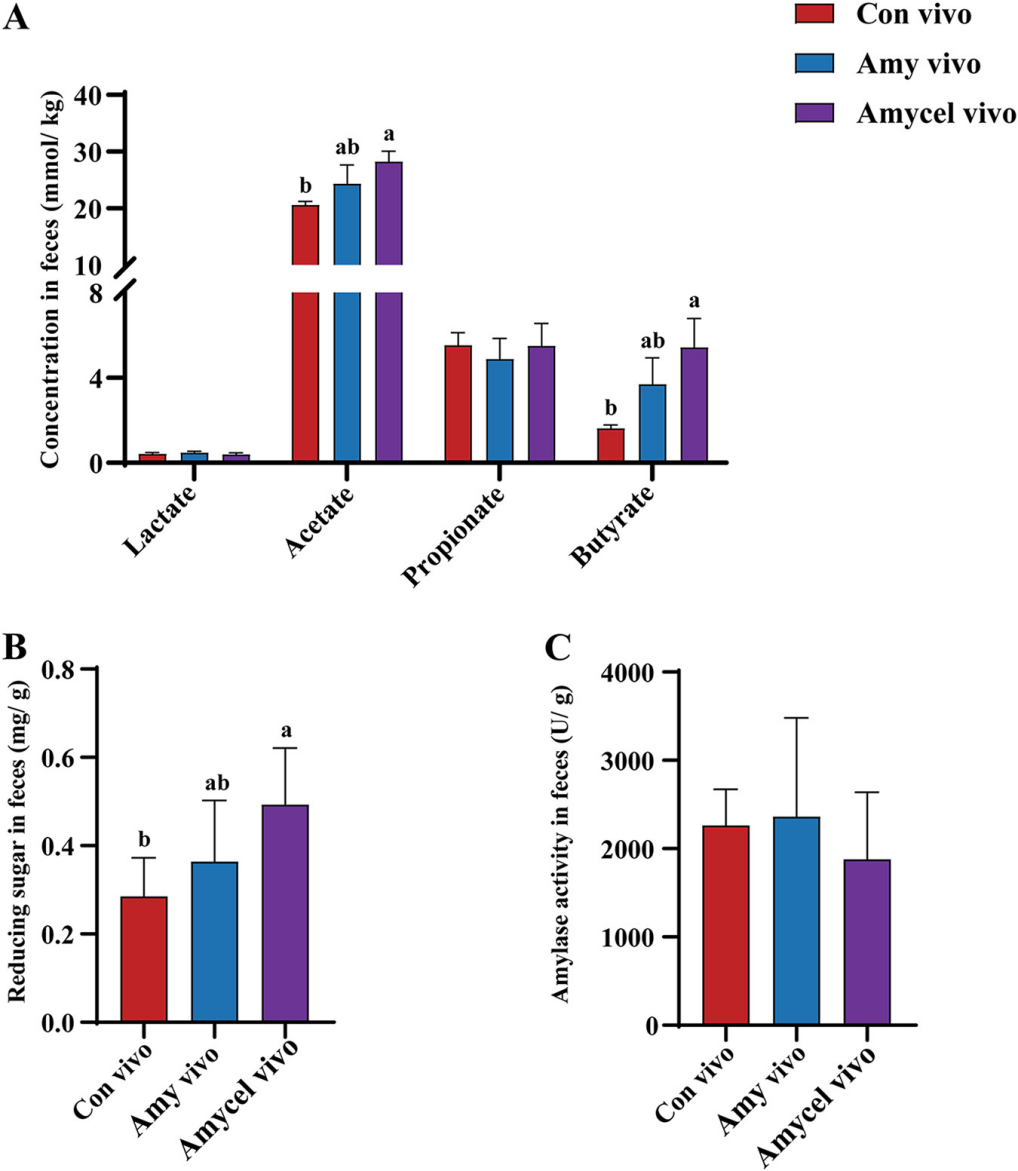
(A) Concentration of lactate, acetate, propionate, and butyrate in feces. (B) Reducing sugar content in feces. (C) Amylase activity in feces. Results are shown as mean ± SEM (Con vivo, *n* = 4; Amy vivo, *n* = 4; Amycel vivo, *n* = 5). Different lowercase letters indicate significant differences, *P < *0.05 (one-way ANOVA with Duncan).

The amylopectin infusion (Amy vivo) did not significantly influence acetate and butyrate production in the hindgut, but the mixture of amylopectin and cellulose infusion (Amycel vivo) significantly increased (*P < *0.05) acetate and butyrate concentration in the hindgut compared with the saline infusion (Con vivo) (Fig. 2A). There was no significant difference in fecal reducing sugar content between the Con vivo and Amy vivo groups, but the Amycel vivo group had a higher fecal reducing sugar content (*P < *0.05) than the Con vivo group (Fig. 2B). Amylase activities in the Con vivo, Amy vivo, and Amycel vivo groups were not significantly different from each other (Fig. 2C).

### α-Diversity, β-diversity, and bacterial composition of fecal microbiota.

**FIG 3:**
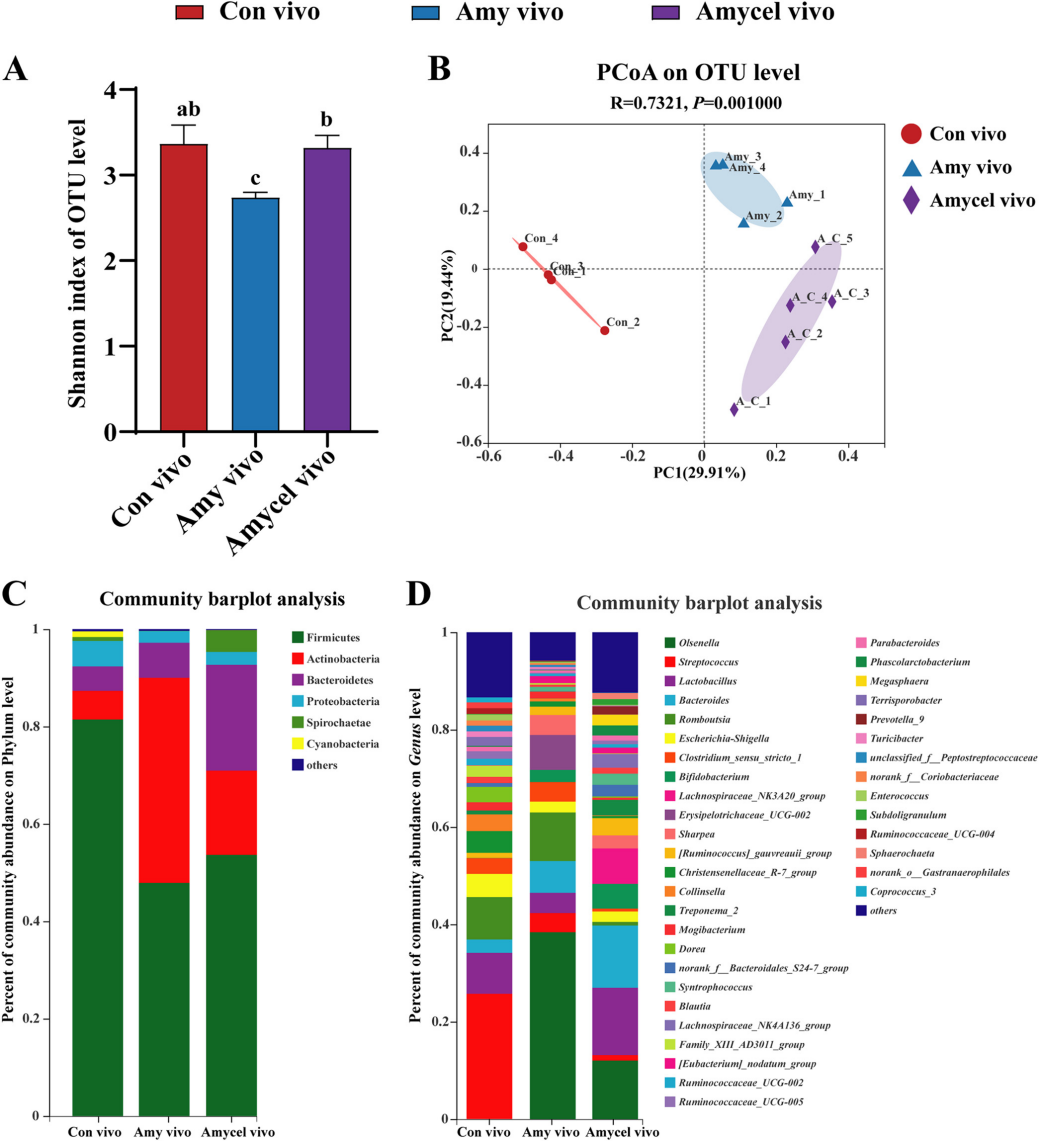
(A) Bacterial α-diversity (Shannon index) of fecal microbiota (Kruskal-Wallis test). (B) Principal coordinate analysis (PCoA) of bacterial communities among all groups (Bray-Curtis distance). Fecal bacteria composition at the phylum (C) and genus levels (D). Results are shown as mean ± SEM (Con vivo, *n* = 4; Amy vivo, *n* = 4; Amycel vivo, *n* = 5). Different lowercase letters indicate significant differences, *P < *0.05.

The mixture of amylopectin and cellulose infusion had a higher (*P < *0.05) Shannon index of fecal microbiota than amylopectin infusion (Fig. 3A). Amylopectin and a mixture of amylopectin and cellulose infusion both significantly changed microbial structure (*R* = 0.7321, *P = *0.001) (Fig. 3B). Firmicutes, Actinobacteria, and Bacteroidetes were the main bacteria in the hindgut: the Amy vivo group had a high relative abundance of *Actinobacteria*, and *Bacteroidetes* was more abundant in the Amycel vivo group than in the other groups (Fig. 3C). At the genus level, *Olsenella* were the main bacteria in the Amy vivo group, *Lactobacillus* and *Bacteroidetes* were at high proportions in the Amycel vivo group (Fig. 3D).

### Bacteria proliferation, microbial functional profiles, and the relationship between differential bacteria and SCFAs.

**FIG 4:**
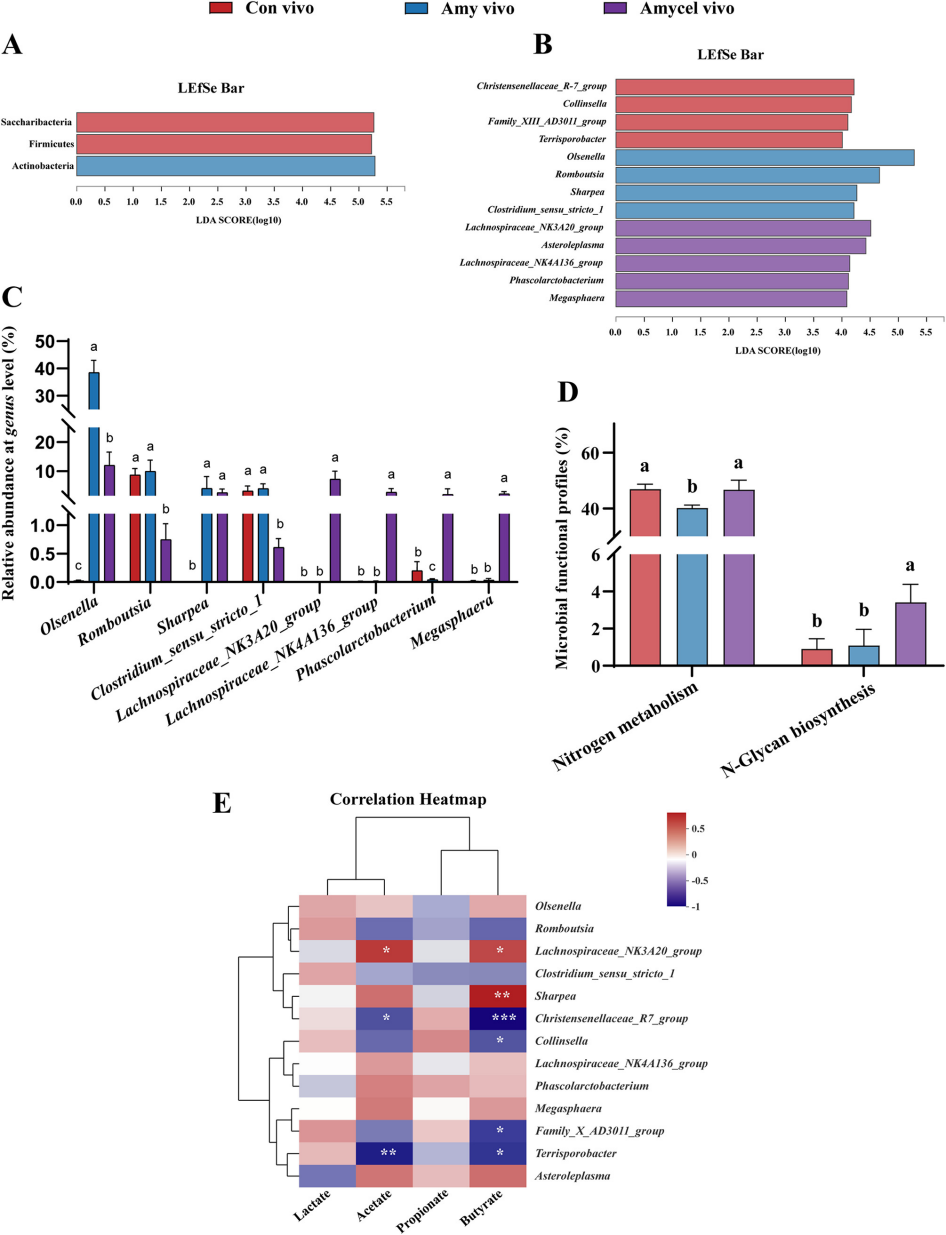
Differential bacteria at the phylum (A) and genus (B) level by linear discriminant analysis. (C) Relative abundance of differential bacteria among all groups (Kruskal-Wallis test). (D) Microbial functional profiles of the fecal microbiota (Kruskal-Wallis test). (E) Correlational relationships between differential bacteria and short-chain fatty acid (SCFA; Spearman’s correlation). Results are shown as mean ± SEM (Con vivo, *n* = 4; Amy vivo, *n* = 4; Amycel vivo, *n* = 5). Different lowercase letters indicate significant differences, *P < *0.05.

A linear discriminant analysis (LDA) effect size (LEfSe) analysis was conducted to distinguish the characteristic intestinal bacteria between different groups, and the results showed that Actinobacteria was significantly enriched in the Amy vivo group (*P < *0.05) (Fig. 4A). *Olsenella*, *Romboutsia*, *Sharpea*, and *Clostridium_sensu_stricto_1* were all significantly enriched in the Amy vivo group (*P < *0.05). The mixture of amylopectin and cellulose infusion significantly promoted the proliferation of *Lachnospiraceae_NK3A20_group*, *Asteroleplasma*, *Lachnospiraceae_NK4A136_group*, *Phascolarctobacterium*, and *Megasphaera* (*P < *0.05) (Fig. 4B). Analysis of differences in relative abundance in characteristic bacteria among all groups showed similar results with LEfSe analysis (Fig. 4C).

The Amycel vivo group had higher (*P < *0.05) nitrogen metabolism ability of fecal microbiota than the Amy vivo group, and fecal microbiota in the Amycel vivo group also had the highest (*P < *0.05) *N*-glycan biosynthesis ability among all groups (Fig. 4D). Results of the relationship between differential bacteria and metabolites showed that *Lachnospiraceae_NK3A20_group* was positively correlated (*P < *0.05) with acetate and butyrate, and *Sharpea* was also positively correlated (*P < *0.05) with butyrate in the hindgut. However, *Christensenellaceae_R7_group* and *Terrisporobacter* were negatively correlated (*P < *0.05) with acetate and butyrate. *Colinsella* and *Family_X_AD3011_group* also had significant negative correlations (*P < *0.05) with butyrate (Fig. 4E).

## DISCUSSION

The starch content (mainly amylopectin) in human and animal diets has increased dramatically during the last few decades. Ingestion of large amounts of starch usually causes undigested amylopectin fermentation in the hindgut and wastes energy (starch fermentation is a low-efficiency means of energy supply) (12). Soluble, high-viscosity fiber is easily wrapped or bonded to amylopectin, preventing contact between amylopectin and amylase and causing amylopectin fermentation in the hindgut (10). However, the mechanisms of amylopectin fermentation and its effects on microbiota and SCFA production in the hindgut required further research. Cellulose has been demonstrated to have a positive effect on probiotic proliferation in the hindgut (13, 14); however, the effects of combined amylopectin and cellulose utilization on hindgut fermentation have rarely been reported.

High vitro DMD and SCFA production was found in the Amy vitro group, but low in the Cel vitro group. Amylopectin could be rapidly fermented by microbes because of its multi-branched-chain structure (branch points at the α-1,6 linkages for every 20 to 25 glucose units) (15). Natural cellulose consists of linear β-1,4-linked d-glucopyranosyl units, and the linear units of cellulose are stabilized by hydrogen bonds between adjacent glucose resides, forming an organized arrangement of cellulose molecules within the microfibrils (16). The substantial difference in structure between starch and cellulose is the main reason for the differences *in vitro* DMD and SCFA production ability during fermentation. One interesting result in this study was that the Amycel vitro group had similar DMD to the Amy vitro group, which was also higher than that of the Cel vitro group. Amylopectin was easily fermented by microbes; microbes proliferated because of amylopectin fermentation also contributed to cellulose degradation. *Bilophila* and *Coprocossus* are amylopectin-degrading microbes and were also reported to have a strong capacity to degrade cellulose (17, 18). The degradation of polysaccharides resulted in an increasing number of reducing ends and reducing sugar content (19). Reducing sugars, including glucose, fructose, and maltose, are carbon sources that can be directly utilized by microbes. Reducing sugar content in the fermentation broth could determine the nutrient supply capacity and the degree of polysaccharide depolymerization (20). Microbes can rapidly convert amylopectin from reducing sugar into SCFA; there was only a small amount of reducing sugar remaining in the fermentation broth. Cellulose could not be fermented rapidly, but it continually produced reducing sugar. Therefore, fermentation of a mixture of amylopectin and cellulose produced the largest amount of reducing sugar. The antioxidant abilities of the Amy vitro and Amycel vitro groups were not significantly different from each other but were higher than that of the control group. Similar levels of SCFA production should be the main reason for this; SCFA have been reported to have strong antioxidant capacity and may be the reason for the high antioxidant ability in the Amy vitro group (21, 22). In this study, the Amy vitro group was demonstrated a preference for propionate production. Strong propionate production ability during starch fermentation was also found in the previous study; the proliferation of propionate-producing bacteria could be the main reason for this (23).

Based on the results of *in vitro* fermentation, we found that the mixture of amylopectin and cellulose had similar fermentation efficiency to amylopectin. However, we needed to elucidate whether the *in vitro* trial results were consistent with the *in vivo* results. The effects and mechanisms of amylopectin and amylopectin partially substituted by cellulose on hindgut fermentation also needed to be determined. Therefore, amylopectin (Amy vivo) and the mixture of amylopectin and cellulose (Amycel vivo) were selected as the substrates for the *in vivo* infusion trial, and a saline infusion (Con vivo) served as a control.

There was no significant difference in fecal reducing sugar content between the amylopectin and saline infusion treatments, but the mixture of amylopectin and cellulose infusion increased fecal reducing sugar content. Except for reducing sugar, the concentration of lactate, acetate, propionate and butyrate in feces were not significantly changed after amylopectin infusion, but the mixture of amylopectin and cellulose infusion significantly promoted acetate and propionate production in the hindgut compared with the Con vivo group. The hindgut is the main site for the fermentation of undigested nutrients; undigested amylopectin is mainly fermented in the cecum and proximal colon. Therefore, amylopectin infusion could not influence reducing sugar content and SCFA concentration in the feces (21). Cellulose has been reported to have a high potential to produce acetate and butyrate during fermentation by promoting the proliferation of acetate- and butyrate-producing bacteria (24, 25); cellulose in the Amycel vivo group was the main reason for the high acetate and butyrate concentration in feces.

Microbes in the hindgut play important roles in energy supplementation, immune regulation, disease prevention, and so on, and hindgut bacterial composition is deeply affected by nutrient substrates (26). Our research found that amylopectin infusion impaired the hindgut microbial Shannon index, but the Shannon index in the Amycel group was significantly higher than that in the Con vivo group. Although rapid amylopectin fermentation in the hindgut resulted in a considerable pH decline and intestinal micro-ecology disturbance, cellulose is a non-starch polysaccharide and has demonstrated the ability to improve microbiota (13). The β-diversity of the Amy vivo and Amycel vivo groups significantly differed from each other. Differential nutrient fermentation causing the β-diversity change has also been proven in previous studies; gut microbe preferences for substrate utilization were the reason for the difference in β-diversity between the Amy vivo and Amycel vivo groups (27, 28).

Firmicutes, Actinobacteria, and Bacteroidetes were the main bacteria in feces at the phylum level and are involved in carbohydrate metabolism and SCFA production (29–31). Amylopectin infusion promoted the proliferation of Actinobacteria, which is one of the four major phyla of the gut microbiota and plays a pivotal role in maintaining gut homeostasis (32). *Bifidobacterium* was the main bacterium in Actinobacteria at the genus level; some *Bifidobacterium* strains are known to effectively degrade starch by attaching to starch particles (33–35). In addition, the relative abundance of *Olsenella*, *Romboutsia*, *Sharpea*, and *Clostridium_sensu_stricto_1* also increased due to amylopectin infusion. *Clostridium_sensu_stricto_1*, *Olsenella*, and *Sharpea* could provide energy for intestinal cells and protect the gut barrier and are seen as butyrate-producing bacteria (36–38). *Romboutsia* is believed to play an important role in gut microbiota dysbiosis and cardiac dysfunction; it has also been positively correlated with indole derivatives, which are harmful metabolites of protein fermentation (39). *Romboutsia* has also been seen as a marker for obesity with different metabolic abnormalities and is positively correlated with serum lipids (including low-density lipoprotein, triglyceride, and total cholesterol) (40). In conclusion, *Romboutsia*, which was abundant in the Amy vivo group, was detrimental to gut microbiota and host metabolism. *Lachnospiraceae_NK3A20_group*, *Asteroleplasma*, *Lachnospiraceae_NK4A136_group*, *Phascolarctobacterium*, and *Megasphaera* were abundant in the Amycel vivo group; *Lachnospiraceae_NK3A20_group* and *Lachnospiraceae_NK4A136_group* were involved in carbohydrate metabolism; and a significant positive correlation was found between acetate, butyrate, and *Lachnospiraceae_NK3A20_group* in this study. A study by Wang et al. also found the ability of *Lachnospiraceae_NK3A20_group* to degrade dietary fiber (41). Another study found that the relative abundance of *Lachnospiraceae_NK3A20_group* was positively correlated with feed efficiency (42). *Lachnospiraceae_NK4A136_group* was also seen as a SCFA-producing bacterium and is significantly correlated with gut barrier function (43). *Phascolarctobacterium* could also promote SCFA production and improve the gut barrier, and its special characteristic of fermenting insoluble dietary fiber has been widely known for decades (44). Therefore, cellulose in the Amycel vivo group could be responsible for *Phascolarctobacterium* proliferation in the hindgut. *Megasphaera*, an important lactate-producing bacterium, is considered an effective endogenous bacterium for preventing acidosis by enhancing nutrient utilization (45). *Megasphaera* also has the potential to ferment undigested protein in the hindgut (46, 47). The nitrogen metabolism ability of microbiota was upregulated after the amylopectin and cellulose mixture infusion, including the degradation of dietary nitrogen and synthesis of microbial protein compounds (48). High nitrogen metabolism activity means high protein utilization ability. *N*-glucan synthesis ability was also strengthened in the Amycel vivo group: *N*-glucan is an important signal molecule in disease regulation and a vital nutrient for hindgut fermentation, which supplies energy for colonic epithelial cells (49, 50). *Christensenellaceae_R7_group* and *Terrisporobacter* were believed to be SCFA-producing bacteria in previous studies (51–53), but they were negatively correlated with acetate and butyrate in this study. *Colinsella* in the hindgut was also negatively correlated with butyrate in this study. Cross-feeding is an important way for microbe colonization in the gut: in this study, foregut bacterial fermentation was absent when substrates were infused into the hindgut, so the lack of foregut bacterial fermentation may be the reason for the negative relationships of *Christensenellaceae_R7_group*, *Terrisporobacter*, and *Colinsella* with butyrate. *Family_X_AD3011_group* also had a negative correlation with butyrate; as a conditionally pathogenic bacterium, it could cause gut microbiota dysregulation or excitation states of other pathogenic bacteria, and it is related to depression and metabolic disturbance (54).

### Conclusion.

The high level of amylopectin fermentation in the hindgut was detrimental to the intestinal microbiota. Partial substitution of amylopectin with cellulose not only enhanced antioxidant ability and fermentation efficiency, but also promoted SCFA production and probiotic colonization in the hindgut.

## MATERIALS AND METHODS

This research was performed following the guidelines of the Laboratory Animal Welfare and Animal Experimental Ethical Inspection Committee at China Agricultural University (AW12601202-1-1) and was approved by the Welfare and Ethics Committee of the Chinese Association for Laboratory Animal Sciences. The animal trial was conducted in the Swine Research Unit of China Agriculture University (Beijing, China).

### *In vitro* fermentation trial.

Amylopectin (98% purity; Macklin, A801574, Shanghai, China) and cellulose used in our previous research (98% purity; Pioneer Biotech Company, Xi’an, China) were selected as the basic substrates in this study (4). The *in vitro* fermentation trial included three groups: amylopectin (Amy vitro; substrate: amylopectin), mixture of amylopectin and cellulose (Amycel vitro; substrate: amylopectin and cellulose, 1:1 [m:m]), and cellulose (Cel vitro; substrate: cellulose). Unlike in the foregut, amylopectin was almost digested during *in vitro* digestion, so the substrates used in this trial were not predigested before fermentation. The *in vitro* fermentation trial protocol was based on previous research with some modifications (55): fresh feces were collected from eight growing pigs (four males and four females, Duroc × Landrace × Large White, 20 to 22 kg), added 10% (m:vol) glycerine and stored in −80°C condition after flash-freezing with liquid nitrogen to conserve bacterial vitality. Pigs were fed a standard corn-soybean meal (Table S1) without antibiotics in the last 3 months before feces collection. Frozen feces were homogenized with 0.1 M phosphate buffer (1:5 m/vol) after thawing, then filled with four layers of sterile gauze to serve as inocula under anaerobic conditions. Next, 100 mg substrate of the three groups was weighed accurately into 6 McCartney bottles and 5 mL inoculum was injected into each; bottles containing inoculum only were set as blank controls (*n* = 6). All bottles were flushed with CO_2_, capped, and incubated for 24 h in a constant temperature incubator shaker (Boxun, Shanghai, China; 39°C at 200 rpm) (56). After incubation, the fermentation broth was centrifuged at 4,000 × *g* for 10 min, and 0.5 mL supernatant was transferred to sterile tubes and stored at −80°C for further analysis. The remaining portions of the fermentation broth were measured for dry matter of unfermented residue after lyophilization.

### *In vivo* ileal infusion trial.

A total of 18 growing pigs (Duroc × Landrace × Large White, 20 ± 1.42 kg) were fitted surgically with a T-cannula in the distal ileum, approximately 5-cm cranial to the ileocecal sphincter, and nursed based on the protocols of previous research (57). All animals were allowed 15 days for surgery recovery and housed in individual stainless-steel metabolism crates (1.4 × 0.9 × 0.9 m) equipped with nipple-drinking devices and feed boxes. Room temperature was maintained at 20 to 25°C throughout the experiment.

The animal experiment lasted for 21 days: 7 days for adaptation and 14 days for ileal infusion. A fiber-free diet (Table 1) was provided throughout the experiment to prevent fiber interference. Diet was divided into two equivalent daily meals and provided at 08:30 and 16:30, diet ingested by pigs exceeded 3 times the estimated requirement for energy maintenance (i.e., 197 Kcal ME/bodyweight kg^0.6^). After the adaptation period, pigs were randomly allocated into three groups (six pigs per group). Pigs in the three groups were infused with 50 mL sterile saline (Con vivo, blank control), 50 mL amylopectin suspension (25 g amylopectin suspended in 50 mL sterile saline; Amy vivo), or 50 mL mixture of amylopectin and cellulose suspension (25 g amylopectin and cellulose mixture suspended in 50 mL sterile saline [amylopectin:cellulose ratio of 1:1], Amycel vivo) through the ileal cannula twice daily (09:00 and 17:00) individually. Unfortunately, the ileal cannula fell off in one pig in the Amycel vivo group and two pigs in the Amy vivo and Con vivo groups during the infusion period. Fresh feces of the remaining pigs were collected by rectal palpation and stored at −80°C for further analysis.

**TABLE 1:** Composition of the growing pigs’ fiber-free diet

Items (as-fed basis)	Content (%)
Corn starch	63.30
Soybean isolated protein	18.40
Soybean oil	3.00
Sucrose	11.30
Lysine	0.20
Methionine	0.10
Threonine	0.10
Limestone	0.30
Dicalcium phosphate	2.40
Salt	0.40
Pre-mix^*a*^	0.50
Total	100.00

a Pre-mix provided the following per kg of complete diet for growing pigs: vitamin A, 5,512 IU; vitamin D3, 2,200 IU; vitamin E, 64 IU; vitamin K3, 2.2 mg; vitamin B12, 27.6 μg; riboflavin, 5.5 mg; pantothenic acid, 13.8 mg; niacin, 30.3 mg; choline chloride, 551 mg; Mn, 40 mg; Fe, 100 mg; Zn, 100 mg; Cu, 100 mg; I, 0.3 mg; Se, 0.3 mg.

### Chemical analysis.

SCFA were measured by ion chromatography according to the methods of Wu et al. (27). Briefly, samples were diluted with ultrapure water filtered through a 0.20-mm Nylon Membrane Filter (Millipore, Bedford, OH), and poured into an ion chromatography system (Dionex ICS-3000, Thermo Fisher Scientific, Waltham, MA, USA). The T-AOC was determined by a total antioxidant capacity assay kit (cat no. A015-1-2; Nanjing Jiancheng Bioengineering Institute, Nanjing, China) based on spectrophotometry. Reducing sugar content was measured by a reducing sugar content assay kit (cat no. BC0030; Solarbio, Beijing, China). Amylase activity was detected by an α-amylase activity detection kit (cat no. BC0615; Solarbio).

### Bacterial community.

Pig feces were tested for the bacteria community, and total microbial genomic DNA was extracted using the QIAamp Fast DNA Stool Minikit (Qiagen, Hilden, Germany) following the manufacturer’s instruction as in a previous report (4). Bacterial 16S rRNA gene fragments (V3-V4) were amplified from the extracted DNA using the primers 338F (5′-ACTCCTACGGGAGGCAGCAG-3′) and 806R (5′-GGACTACHVGGGTWTCTAAT-3′). Low-quality reads were removed by PANDAseq (version 2.9) (58), and the high-quality sequences were clustered into operational taxonomic units (OTUs) with 97% similarity using UPARSE (version 7.0) in QIIME (version 1.8) (59, 60). Taxonomy was assigned to OTUs using the RDP classifier against the SILVA 16S rRNA gene database (release 128^2^) with a confidence threshold of 70%. α-Diversity was evaluated by calculating the Shannon index with the mothur program (version 1.30.1) (61). Principal coordinate analysis (PCoA) was performed based on the Bray-Curtis distance, and an analysis of similarity (ANOSIM) based on Bray-Curtis distance was performed to compare the similarity of the microbial community, and PICRUSt2 was used to analyze microbial functional profiles.

### Statistical analysis.

The data were analyzed using the SPSS software package (SPSS version 20.0, SPSS Inc., Chicago, IL, USA), and statistical variations were estimated by the standard error of the means. Microbial functional profile differences among groups were determined using Kruskal-Wallis test. The characteristic bacteria of different groups were classified by using the LEfSe analysis (significant when LDA > 4.0). In other analyses, a one-way analysis of variance with Duncan was used to determine statistical differences. Correlations between SCFA and differential bacteria were analyzed by Spearman’s correlation. All statistical analyses were considered significant at *P < *0.05.

### Data availability.

All microbial sequence data have been uploaded to NCBI (PRJNA752810).
