# Supplementary material for: Amylopectin Partially Substituted by Cellulose in the Hindgut Was Beneficial to Short-Chain Fatty Acid Production and Probiotic Colonization
Source: Microbiol Spectr. 2023 Apr 10;11(3):e03815-22. doi: 10.1128/spectrum.03815-22 (PMC10269567; doi:10.1128/spectrum.03815-22)
Supplement: Supplemental file 1 — Table S1. Download spectrum.03815-22-s0001.pdf, PDF file, 0.1 MB [file spectrum.03815-22-s0001.pdf]

Supplemental Table 1. Ingredients and chemical composition of growing pig's diet.

Items (%, as-fed basis)	Content
Ingredients	
Corn	72.50
Soybean meal	25.00
Limestone	0.75
Dicalcium phosphate	0.90
Salt	0.35
Premix[1]	0.50
Chemical composition, % DM basis	
DM	88.17
Crude protein	16.62
Exther extract	2.42
Starch	46.26
Neutral detergent fiber	11.02
Acid detergent fiber	3.06
Ash	4.08

[1]Premix provided the following per kg of complete diet for growing pigs: vitamin A, 5,512 IU; vitamin D3, 2,200 IU; vitamin E, 64 IU; vitamin K3, 2.2 mg; vitamin B12, 27.6 ug; riboflavin, 5.5 mg; pantothenic acid, 13.8 mg; niacin, 30.3 mg; choline chloride, 551 mg; Mn, 40 mg; Fe, 100 mg; Zn, 100 mg; Cu, 100 mg; I, 0.3 mg; Se, 0.3 mg.
